# Solid-state ^31^P and ^1^H chemical MR micro-imaging of hard tissues and biomaterials with magic angle spinning at very high magnetic field

**DOI:** 10.1038/s41598-017-08458-0

**Published:** 2017-08-15

**Authors:** Maxime Yon, Vincent Sarou-Kanian, Ulrich Scheler, Jean-Michel Bouler, Bruno Bujoli, Dominique Massiot, Franck Fayon

**Affiliations:** 1 CNRS, CEMHTI UPR3079, Université d’Orléans, F-45071 Orléans, France; 2 Leibniz-Institut für Polymerforschung Dresden e.V., Hohe Str. 6, Dresden, Germany; 3 CEISAM, Université de Nantes, CNRS, 2 rue de la Houssinière, BP 92208, 44322 Nantes, Cedex 3 France

## Abstract

In this work, we show that it is possible to overcome the limitations of solid-state MRI for rigid tissues due to large line broadening and short dephasing times by combining Magic Angle Spinning (MAS) with rotating pulsed field gradients. This allows recording *ex vivo* ^31^P 3D and 2D slice-selected images of rigid tissues and related biomaterials at very high magnetic field, with greatly improved signal to noise ratio and spatial resolution when compared to static conditions. Cross-polarization is employed to enhance contrast and to further depict spatially localized chemical variations in reduced experimental time. In these materials, very high magnetic field and moderate MAS spinning rate directly provide high spectral resolution and enable the use of frequency selective excitation schemes for chemically selective imaging. These new possibilities are exemplified with experiments probing selectively the 3D spatial distribution of apatitic hydroxyl protons inside a mouse tooth with attached jaw bone with a nominal isotropic resolution nearing 100 µm.

## Introduction

Magnetic Resonance Imaging (MRI) is a well-known non-ionizing powerful tool allowing the imaging of soft tissues with wide range of applications in clinical medicine. For these applications, MRI relies on the observation of mobile molecular species in the tissues using the intense and narrow resonance of an abundant NMR-active isotope, typically the ^1^H signal of free water molecules. In such a case, the method benefits from a high NMR-signal receptivity and from long coherence lifetimes allowing spatial encoding with spin echo or gradient echo sequences^1–3^. For rigid materials, the situation is different and the spatial resolution and sensitivity are limited by the strong line broadenings due to the presence of large anisotropic nuclear spin interactions, like dipolar couplings or chemical shift anisotropy (CSA), and magnetic susceptibility effects^4^. Strong dipolar couplings typical of the solid-state also usually lead to short dephasing times (T_2_’) making conventional spin echo MRI sequences inefficient, while in frequency-encoded imaging the spatial resolution remains limited by the anisotropic line broadenings^5^. The combination of these effects make challenging the improvement of both spatial resolution and signal to noise ratio (SNR) in MR images of rigid solids^6, 7^.

Despite its intrinsic difficulties, solid-state MRI has attracted a significant interest for *in-vivo* and *in-vitro* studies of hard tissues and related biomaterials. Indeed, the various possibilities of contrast in solid-state MRI appeared highly complementary to X-ray micro-computed tomography and liquid state MRI, which are usually used to obtain direct and negative high-resolution images of calcified tissues, respectively^8, 9^. Several works have thus been devoted to the use of solid-state MRI for the imaging of bones and teeth with a sub-millimeter spatial resolution^10–35^. Most of these works used the broad ^1^H resonances of water molecules bound to the mineral or to the collagen matrix, or in the small pore of cortical bones for imaging rigid tissues with efficient contrast^10–25^. Alternatively, ^31^P solid-state MRI was early proposed as direct probe of mineral phase (hydroxyapatite) in rigid tissues and for the imaging of synthetic calcium phosphate implants used for bone repair^26, 27^. However due to the lower gyromagnetic ratio of ^31^P relative to ^1^H, spatial resolution remained limited (∼0.5 to 1 mm) and only few studies have recently employed ^31^P MRI to obtain *in vivo* or *ex vivo* quantitative information about the degree of mineralization in rigid tissues^28–35^.

To overcome the drawbacks associated to strong line broadening and very short dephasing times of the ^1^H and ^31^P signals of rigid tissues, previous works employed strong magnetic field gradients dominating the natural NMR line width with consecutive excitation-detection schemes, like ZTE^36^, UTE^37^, STRAFI^38^ and STRAFI-MAS^39, 40^ or SWIFT^41^ methods. Nevertheless, the anisotropic line broadening combined with strong magnetic field gradients decreases the sensitivity of the experiment, thereby limiting the SNR and spatial resolution. As illustrated in seminal work^42^, multiple-pulse line narrowing techniques are an alternative option to decrease line broadening effects in solid-state MRI. For example, multiple-pulse homonuclear decoupling sequences^43^ or magic sandwich echoes^44, 45^ have been combined with pulsed field gradients to improve ^1^H solid-state polymer micro-imaging^43–46^ and, more recently, the quadratic echo line narrowing method was introduced for ^31^P imaging of rigid tissues with enhanced spatial resolution^47^. Magic Angle Spinning (MAS), which is the key stone of the high-resolution solid-state NMR methods, was also early combined with magnetic field gradients to obtain MR images of solids affected by significant line broadenings^48–50^. However, despite its capabilities for MR micro-imaging^51, 52^ and spatially resolved solid-state^13^C NMR spectroscopy^53, 54^ in the case of polymer materials, the applications of solid-state MAS MRI were rarely investigated for the *ex vivo* characterization of the inorganic microstructure of rigid tissues.

In this work, we investigate the use of Magic Angle Spinning for *ex vivo* micro-imaging applications allowing ^31^P and ^1^H 3D visualization of the inorganic content of rigid tissues with enhanced spatial (and spectral) resolution. Since the ^31^P and ^1^H spectra of the apatite crystals in bones and teeth, similarly to other orthophosphates, are subjected to moderate dipolar interactions and weak chemical shift anisotropy effects, moderate spinning frequencies (5–10 kHz) allow efficient line narrowing^55, 56^. Hence, the spinning rate can be limited to avoid potential undesirable effects due to centrifugal forces, while keeping benefit of MAS. In such cases, increasing the magnetic field can lead to sensitivity enhancement without increasing the anisotropic broadenings. Here, we thus use the combination of MAS at very high magnetic field (17.6 T) with rotating pulsed field gradients to obtain ^31^P 2D slice-selected and 3D solid-state images with highly enhanced SNR and spatial resolution. Solid-state NMR methods such as cross-polarization (CP) MAS are shown to offer additional contrast in the obtained ^31^P images and to reduce the experiment duration. It is shown that MAS at very high field gives high-resolution ^1^H spectra and the possibility of ^1^H chemically-selective 3D imaging in rigid tissues using selective spin echo MAS MRI sequences is also demonstrated, through the 3D quantitative visualization of the hydroxyl content in a mouse tooth and jaw bone sample.

## Materials and Methods

The ^31^P and ^1^H solid-state NMR and MRI experiments were carried out on a wide-bore (89 mm) Advance III HD Bruker spectrometer operating at a magnetic field of 17.6 T (^1^H and ^31^P Lamor frequencies of 750.1 and 303.6 MHz, respectively) and equipped with a MAS probe fitted in a Bruker Micro 2.5 gradient system delivering magnetic field gradients of 2.5 Gauss.cm^−1^.A^−1^ along the three axis of the Laboratory Frame (LF). The maximum gradient strength was 100 Gauss.cm^−1^ per axis (maximum current intensity of 40 A). All experiments were performed using a Bruker 3.2 mm double resonance MAS probe (maximum spinning frequency of 24 kHz). The samples were packed into rotors with internal diameter and length of 2.6 and 8 mm.

In this setup, the gradient z-axis is collinear to the principal magnetic field B_0_ and the probe is oriented such that the projection of the MAS axis in the plane perpendicular to B_0_ bisects the x and y LF gradient axis^57^. To obtain images of objects rotating at the magic angle, the gradients must be applied in the MAS rotating frame. The expressions of gradients in the MAS frame as a function of the LF gradients are directly obtained from the rotation matrix with Euler angles (45°, θ_M_, ω_R_t) defining the relative orientations of the two different orthogonal axis system, where θ_M_ is the magic angle (54.74°) and ω_R_ is the spinning frequency^57^. Accordingly, a gradient along the MAS axis, which is defined as the z^MAS^ direction, is obtained from a linear combination of the three LF gradients:

1 $${{\rm{G}}}_{{\rm{z}}}^{{\rm{MAS}}}=\frac{1}{\sqrt{3}}{{\rm{G}}}_{{\rm{x}}}^{{\rm{LF}}}+\frac{1}{\sqrt{3}}{{\rm{G}}}_{{\rm{y}}}^{{\rm{LF}}}+\frac{1}{\sqrt{3}}{{\rm{G}}}_{{\rm{z}}}^{{\rm{LF}}}$$

The gradients along the x^MAS^ and y^MAS^ axis are obtained from combinations of amplitude-modulated LF gradients synchronized with the spinning frequency and are given by:

2 $$\begin{array}{rcl}{{\rm{G}}}_{{\rm{x}}}^{{\rm{MAS}}} & = & (\frac{1}{\sqrt{6}}\,\cos ({{\rm{\omega }}}_{{\rm{R}}}{\rm{t}})-\frac{1}{\sqrt{2}}\,\sin ({{\rm{\omega }}}_{{\rm{R}}}{\rm{t}})){{\rm{G}}}_{{\rm{x}}}^{{\rm{LF}}}\\  &  & +\,(\frac{1}{\sqrt{6}}\,\cos ({{\rm{\omega }}}_{{\rm{R}}}{\rm{t}})+\frac{1}{\sqrt{2}}\,\sin ({{\rm{\omega }}}_{{\rm{R}}}{\rm{t}})){{\rm{G}}}_{{\rm{y}}}^{{\rm{LF}}}-\frac{1}{\sqrt{3}}\,\cos ({{\rm{\omega }}}_{{\rm{R}}}{\rm{t}}){{\rm{G}}}_{{\rm{z}}}^{{\rm{LF}}}\end{array}$$

3 $$\begin{array}{rcl}{{\rm{G}}}_{{\rm{y}}}^{{\rm{MAS}}} & = & (-\frac{1}{\sqrt{2}}\,\cos ({{\rm{\omega }}}_{{\rm{R}}}{\rm{t}})-\frac{1}{\sqrt{6}}\,\sin ({{\rm{\omega }}}_{{\rm{R}}}{\rm{t}})){{\rm{G}}}_{{\rm{x}}}^{{\rm{LF}}}\\  &  & +\,(\frac{1}{\sqrt{2}}\,\cos ({{\rm{\omega }}}_{{\rm{R}}}{\rm{t}})-\frac{1}{\sqrt{6}}\,\sin ({{\rm{\omega }}}_{{\rm{R}}}{\rm{t}})){{\rm{G}}}_{{\rm{y}}}^{{\rm{LF}}}+\frac{1}{\sqrt{3}}\,\sin ({{\rm{\omega }}}_{{\rm{R}}}{\rm{t}}){{\rm{G}}}_{{\rm{z}}}^{{\rm{LF}}}\end{array}$$

Using these gradient shapes and fixing the rotor pitch allow performing Magic Angle Spinning MRI. In the 2D and 3D spin-echo MRI pulse sequences employed here, the rotor pitch (electronically monitored) triggers the first excitation pulse. The echo delay and the time between the start of read dephase and read gradients were set to an integer number of rotor periods to observe in-phase spinning sidebands (if present) and to preserve sample orientation between read-dephase and read gradients. It should be noted that the rotor-frame gradient shapes require the three LF gradient coils to deliver the same gradient strengths along the MAS axis. The currents applied to the three coils were thus carefully adjusted by maximizing the center band intensity of the 1D profile of a rotating cylindrical silicone sample when applying combinations of two (of the three) LF gradients. Maximum intensity was obtained when the resulting gradient was perpendicular to the MAS axis (corresponding to equivalent intensities of the two applied gradients). Experimentally, it was observed that the amplitudes of the x^MAS^ and y^MAS^ amplitude-modulated gradients decrease slightly when increasing the rotor spinning frequency^57^. This variation of gradient amplitudes with the spinning frequency was measured on a cylindrical silicone phantom and fitted with a polynomial function to calculate a current correcting factor accounting for this effect. This correcting factor was 1.44 at a spinning rate of 10 kHz. As commonly employed, we use a trapezoidal shape for better stabilization of all gradient pulses. The employed MAS frequency (10 kHz) is related to a time constant (rotor period) of 100 μs and is within the range of the requirement for micro-imaging gradient switching time, the effects of which are minimized for a sinusoidal amplitude modulation. The gradient coils are self-shielded to minimize the effective gradient outside the coil. Effects related to residual eddy current inside the probe were not noticed. The nominal spatial resolution, which should not be confused with the often quoted pixel resolution, is defined in the frequency encoding (read) dimension as^7^:

4 $$\Delta x=\,\frac{\Delta \nu }{\gamma G}\,$$

where Δν, γ and G correspond to the full width at half maximum (fwhm) of the resonance, the gyromagnetic ratio of the observed nucleus and the gradient strength, respectively.

The nominal spatial resolution in the phase encoding dimension is defined as^7^:

5 $$\Delta x=\,\frac{1}{2\gamma {G}_{max}\delta }$$

where γ is the same as above, G_max_ is the maximum strength of the phase incremented gradient and δ is the duration of the square equivalent gradient.

All pulse programs and automation programs setting up the parameters were developed on Topspin 3.2. Selection of the desired coherence transfer pathway in spin echo experiments was done using a 16-step nested phase cycle. ^1^H- ^31^P CPMAS spin echo MRI experiments were performed at a spinning frequency of 10 kHz using ^31^P and ^1^H nutation frequencies of about 40 and 50 kHz, respectively. For the 2D image with direct excitation, the ^31^P RF-field strength was 50 kHz. The 3D ^1^H spin echo MAS were obtained at a spinning frequency of 10 kHz. The ^1^H nutation frequency was 50 kHz. ^31^P or ^1^H frequency-selective refocusing-SNOB^58^ pulses were used for slice selection. Additional experimental parameters are given in figure captions. Processing of the datasets was also performed with Topspin 3.2. All datasets were zero-filled prior to Fourier transform. No apodisation was applied except for the ^31^P 3D MAS MRI image of the mouse tooth, for which a Hann apodization function was used. Image display and manipulation was done with Matlab® R2013b. The signal to noise ratio (SNR) of the 2D and 3D images is defined as the maximal signal intensity divided by the standard deviation of the noise.

The X-Ray micro computed tomography image was recorded using a Bruker-microCT SkyScan 1272. Scan images were acquired with the following parameters: tube voltage 60 kV, tube current 166 μA, exposure time 500 ms, rotation step 0.45°, covered angle 188.55°, voxel size 4 × 4 × 4 μm^3^, FOV 4 × 4 × 4 mm^3^, frame averaging 2, acquisition time 16 min.

The calcium-deficient hydroxyapatite nanocristalline powder was obtained by alkaline hydrolysis of dicalcium phosphate dihydrate using aqueous ammonia^59^. The α-tricalcium phosphate sample was prepared by calcination of a 2:1 molar mixture of CaHPO_4_ and CaCO_3_ at 1350 °C for at least 4 hours, and subsequent rapid cooling to room temperature. The stoichiometric hydroxyapatite sample (99.9%) was purchased from Sigma Aldrich. The mouse tooth sample corresponds to a molar attached to a piece of jaw bone, which was allowed to dry under room atmosphere over two years without any further specific treatment. The tooth sample was centered and held in the rotor by compacted alumina powder (Figure S1) to spread the physical constrains on the whole sample and allow good balancing of the rotor.

## Results and Discussion

The potential of magic angle spinning (MAS) MRI at very high magnetic fields for obtaining *ex-vivo* ^31^P images of rigid tissues and biomaterials was first investigated on a phantom of calcium-deficient hydroxyapatite (CDA). It consists of a Kel-F insert with three cylindrical boreholes, two parallel and one perpendicular to the spinning axis (1 mm in diameter), which were filled with a powder of CDA nanoparticles (Fig. 2a). The structure of such CDA nanoparticles (Ca/P = 1.56, needle shape, length∼45 nm) was investigated in details by high-resolution solid-state NMR^60, 61^. They are made of an ordered apatitic core with a disordered outer layer containing non-apatitic HPO_4_ groups, adsorbed water molecules and deficient in hydroxyl ions. In that respect, the CDA nanoparticles show similarities with what reported for the apatite crystals in bones^62^.

**Figure 1:**
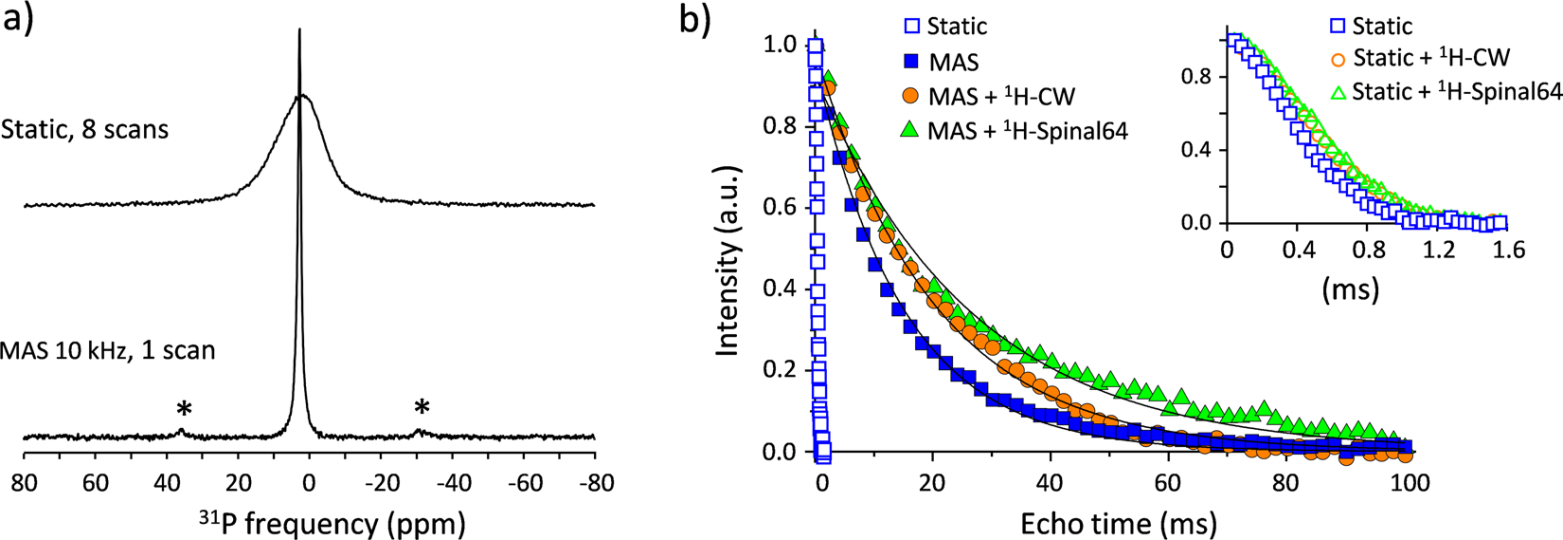
(**a**) ^31^P NMR spectra of the CDA phantom recorded at 17.6 T without (top, 8 scans) and with MAS (bottom, one scan) at a spinning frequency of 10 kHz. The asterisks indicate spinning sidebands. (**b**) ^31^P spin-echo decay curves of the CDA phantom without MAS (open blue squares) and with MAS (filled blue squares) at a spinning frequency of 10 kHz. The MAS spin echo decay curves obtained applying ^1^H CW or ^1^H SPINAL64 decoupling (^1^H RF-field strength of 100 kHz) during the echo time are shown as filled orange circles and filled green triangles, respectively. The black lines correspond to best fits of experimental data with single exponential decay functions. The corresponding T_2_’ values are 15, 20 and 26.7 ms, without ^1^H decoupling, with CW and with SPINAL64 decoupling, respectively. The insert shows a zoom of the ^31^P spin echo decay under static conditions without ^1^H decoupling (open blue squares), and with CW (open orange circles) or SPINAL64 decoupling (open green triangles).

The ^31^P spectra of the CDA phantom recorded at very high magnetic field (17.6 T) without and with MAS are shown in Fig. 1. As expected, MAS provides efficient line narrowing and leads to significant increase of both spectral resolution and SNR. Using a spinning frequency of 10 kHz at a field of 17.6 T, the ^31^P linewidth is decreased from 4700 to 340 Hz. A nearly isotropic spectrum is obtained with one intense resonance (at 2.8 ppm), very weak residual sideband intensities (below 2% of the total intensity) and a ∼11 times larger SNR. For calcium orthophosphates (like CDA, hydroxyapatite or tricalcium phosphate), the ^31^P chemical shift anisotropy (CSA) is small^63, 64^ and the line broadening is mainly due to moderate dipolar interactions. In such case, sideband-free ^31^P MAS spectra can be obtained under moderate spinning conditions even using a very high magnetic field. Using faster spinning rates or applying ^1^H decoupling during signal acquisition did not further decrease the residual linewidth. For CDA, this MAS linewidth is mainly due to a distribution of ^31^P isotropic chemical shift which reflects the structural disorder in the nanoparticles (as for the apatite crystals in bone and tooth) and, consequently, it increases nearly linearly with the magnetic field B_0_ (from 180 Hz at 9.4 T to 340 Hz at 17.6 T). As well, increasing the magnetic field leads to an increase of the ^31^P longitudinal relaxation time constant (Figures S2 and S3). In the case of the CDA and for the tooth sample studied hereafter, the effect on sensitivity of the increase with B_0_ of both ^31^P MAS linewidth and longitudinal relaxation time is countered by the field-induced increase of the sensitivity (proportional to B_0_^3/2^). This results in a slight increase of the SNR ratio in ^31^P MAS spectra (in the range 5–10% from 9.4 to 17.6 T). However, this situation may not be considered as general case since the ^31^P relaxation time and its field-dependence could vary as a function of apatitic hydroxyl and water content. It should also be noted that as the ^31^P linewidth increase with B_0_, frequency encoding gradient have to be increased proportionally to achieve equivalent spatial resolution.

MAS also leads to a significant increase of the ^31^P transverse dephasing time (T_2_’) measured in a Hahn echo experiment. Without MAS, the ^31^P signal shows a fast dephasing with a non-exponential decay due to dipolar effects, and half of the ^31^P magnetization is lost after an echo time (TE) of 400 µs. At a MAS frequency of 10 kHz, a much slower exponential decay of the echo intensity is observed (T_2_’ = 15 ms) and 85% of the ^31^P signal remains for an echo time of 2.5 ms. As mentioned above, ^1^H decoupling does not reduce the inhomogenously broadened ^31^P linewidth, but it can be employed to further increase coherence lifetimes^65^. For example, applying continuous wave or SPINAL64 ^66^ decoupling with 100 kHz RF-field strength leads to longer ^31^P dephasing time (T_2_’) up to 20 or 26.7 ms, respectively (Fig. 1b).

MAS alone thus provides efficient ^31^P line narrowing and extended dephasing time in calcium orthophosphates samples, allowing the use of spin-echo sequences for ^31^P solid-state imaging. To directly obtain images of the sample rotating at the magic angle, the effective gradients must be applied along the principal axes of the rotor frame and synchronized with the sample spinning^49, 50^. As described in the experimental section, gradient along the rotor axis (z^MAS^) is obtained using a linear combination of three orthogonal LF gradients of constant amplitudes, while those along x^MAS^ and y^MAS^ rotating axes result from sinusoidal amplitude-modulated gradients synchronized with the spinning frequency^57^.

**Figure 2:**
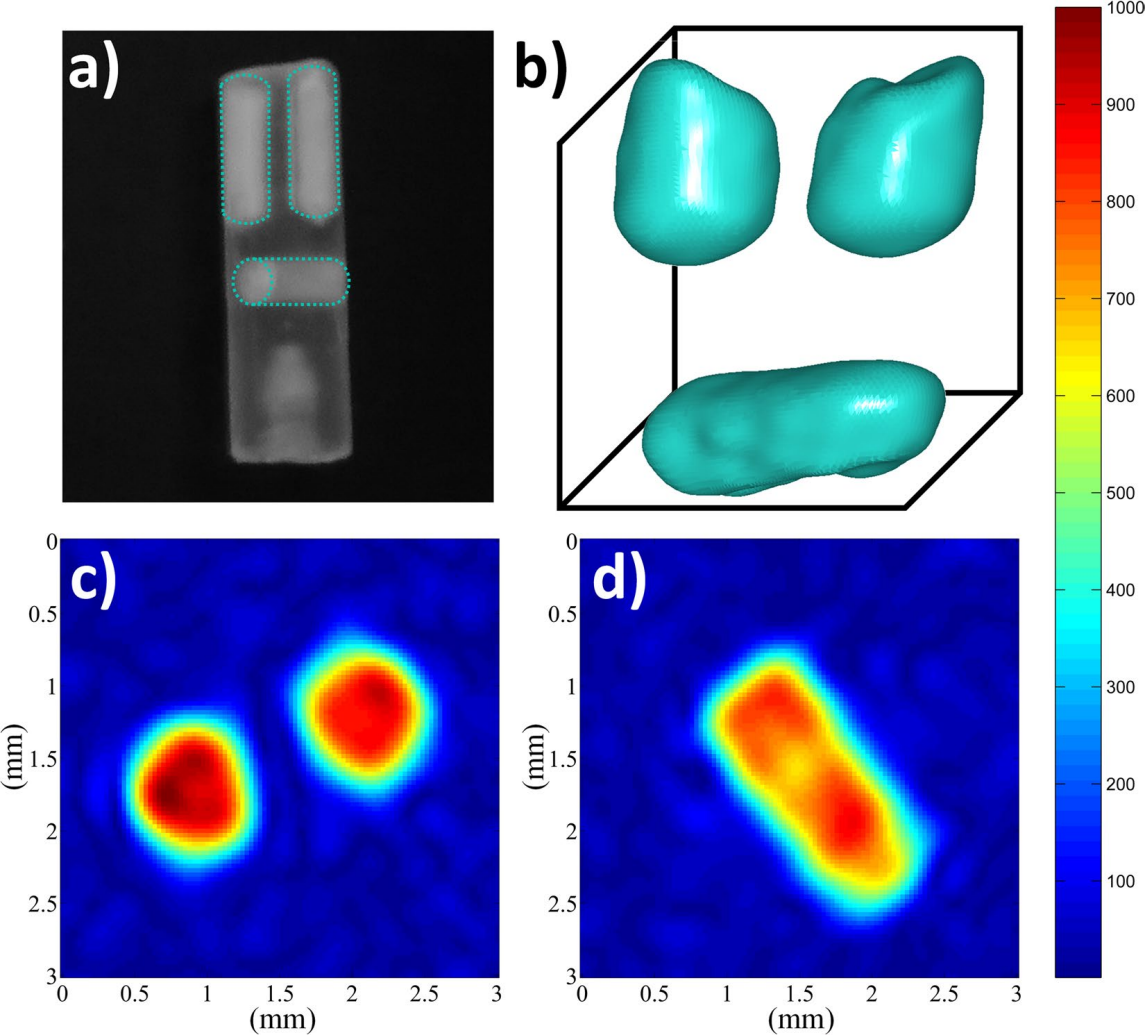
(**a**) Picture of the phantom made of a PCTFE (Kel-F) insert with two boreholes parallel and one perpendicular to the spinning axis (1 mm in diameter) filled with the CDA powder. (**b**) Isosurface representation of the ^31^P 3D MAS image of the phantom recorded at 17.6 T with a spinning frequency of 10 kHz using the spin echo sequence shown in Fig. 3a. The isosurface value is 40% of the maximum signal intensity. ^1^H-^31^P cross polarization with a contact time of 3 ms was used and 80 transients were co-added with a recycle delay of 0.5 s corresponding to an experimental duration of 11 h 32 min. The nominal resolution is 132 µm in the read dimension (corresponding to a gradient of 15.47 G/cm) and 155 µm in the phase dimensions with a field of view of 7.5*7.5*15 mm. SNR = 103. The dataset was zero-filled three times leading to voxel size of 30 × 30 × 30 µm^3^. (**c**,**d**) ^31^P 2D slice-selected MAS images of the CDA phantom taken at (**c**) the top and d) the middle part of the sample. A refocusing-SNOB pulse of 900 µs duration (excitation bandwidth of 2.6 kHz) was used to select a slice of 710 µm thickness. The spatial resolution is 117 µm in read dimension (gradient of 17.44 G/cm) and 155 µm in phase dimension, corresponding to an experimental time of 17 min. SNR = 60 and pixel size of 30 × 30 µm^2^ after zero-filling of the dataset. The color bar scales from zero up to the maximum signal intensity.

The ^31^P image of the CDA phantom recorded with this approach using a 3D spin echo MRI sequence at MAS frequency of 10 kHz is shown in Fig. 2. The image nicely reproduces the sample geometry without any visible blurring effects related to the presence of the weak intensity residual sidebands in the MAS spectrum. The image was obtained with an echo time (TE) of 2.8 ms for which 83% of the ^31^P signal was refocused. To increase the efficiency of the MRI experiment, solid-state ^1^H- ^31^P cross-polarization (CP) MAS was used with a contact time of 3 ms. Because ^31^P has much longer longitudinal relaxation times than ^1^H, CPMAS also allows using much shorter repetition times (TR) than with direct ^31^P excitation. Despite CP leads to lower ^31^P signal intensity for the CDA sample (24% of that obtained with direct excitation using a 90° flip angle), it provides this way a significant reduction of the experiment duration and enables recording the ^31^P 3D spin echo MAS image of the phantom with a nominal spatial resolution of 132 × 155 × 155 µm^3^ and a SNR of 103 in 11.5 hours. It should be noted that other polarization transfer methods can be employed and, for example, ^31^P signal enhancement (up to 20%) using ^1^H- ^31^P nuclear Overhauser effect in static solid-state MRI was recently reported^35^. However, the efficiency of ^1^H- ^31^P polarization transfer methods will strongly depends on the hydroxyl and water content of the apatite structure.

**Figure 3:**
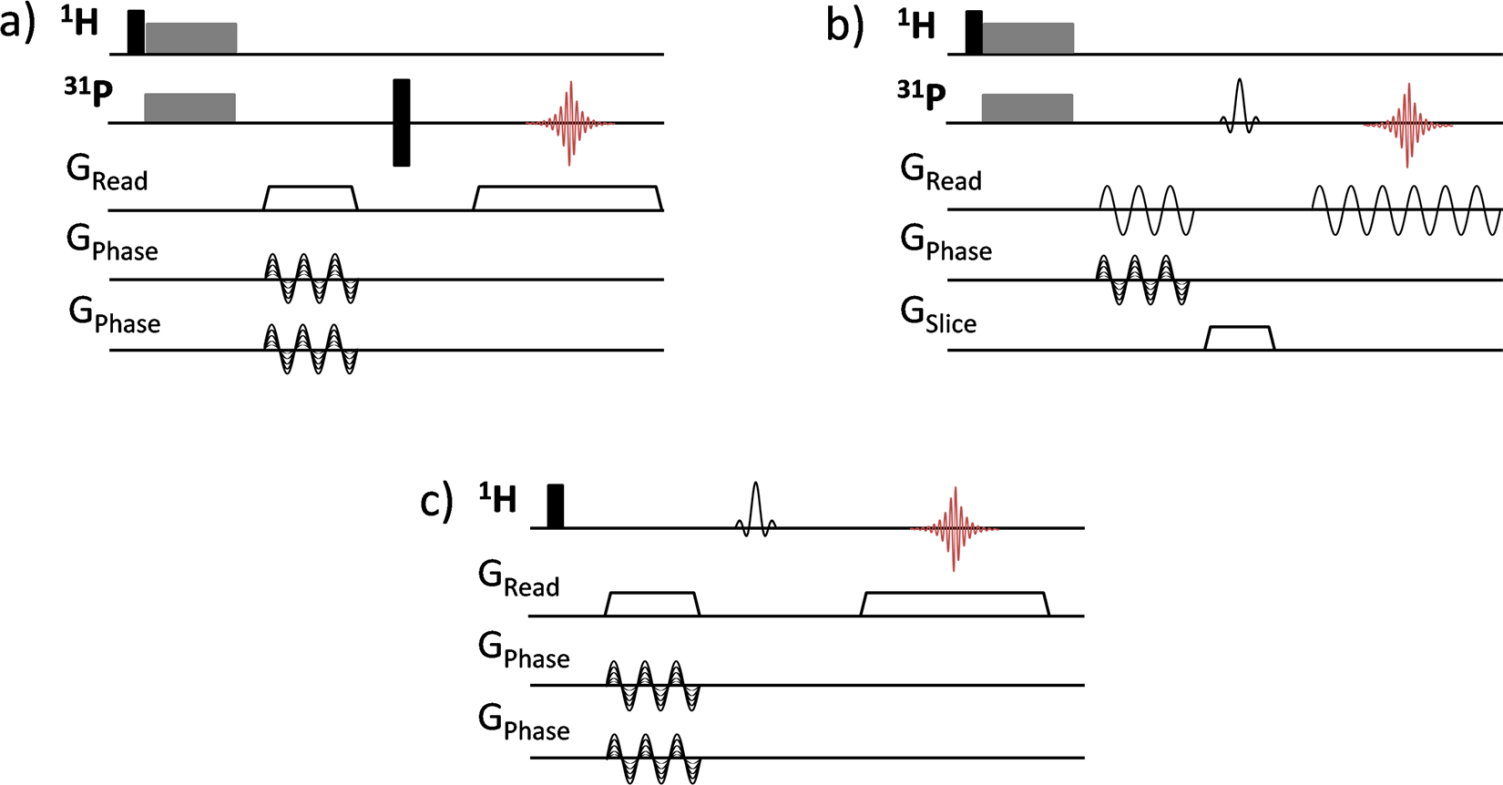
MRI spin-echo sequences used for magic angle spinning (**a**) 3D ^31^P CPMAS imaging, (**b**) 2D ^31^P CPMAS slice-selective imaging and (**c**) 3D ^1^H MAS chemically-selective imaging. Black narrow rectangles correspond to 90° and 180° hard pulses, spin lock block of CP transfer are indicated as grey wide rectangles. The gradient pulses along the MAS axis (z^MAS^) are represented as trapezoids and the rotating gradients along the x^MAS^ and y^MAS^ axes, composed of LF gradients with sinusoidal amplitude modulations, are depicted as sinusoidal curves.

The large increase of the ^31^P coherence lifetimes under MAS makes also possible to use larger TE and a selective refocusing pulse of longer duration and narrow bandwidth, as required for ^31^P slice-selection imaging. As an illustration, ^31^P MAS 2D slice-selection images of the top and bottom parts of the phantom, with slice thicknesses of 710 µm, are shown in Fig. 2. As for the 3D spin echo experiment, ^1^H-^31^P CPMAS was used to reduce experimental duration and each 2D image with a spatial resolution of 117 × 155 µm^2^ was obtained in 17 min. It should be noted that the employed spin-echo MAS sequence with slice-selection along the MAS axis involves a frequency encoding step with amplitude-modulated rotating gradients (Fig. 3b). Frequency encoding under MAS with a rotating gradient is known to be sensitive to experimental imperfections and, in particular, a slight mismatch between the rotor position and the center of the magnetic field gradients can give rise to sidebands images^67^, as reproduced from numerical simulations (not shown). In such case, the gradient strength has to be limited to avoid the overlap of the image with the first spinning sideband images that would lead to distortion effects. In that respect, the 3D spin echo sequence which uses a non-modulated gradient for frequency encoding along the z^MAS^ axis and rotating gradients for phase encoding appears much more robust to such an experimental imperfection.

**Figure 4:**
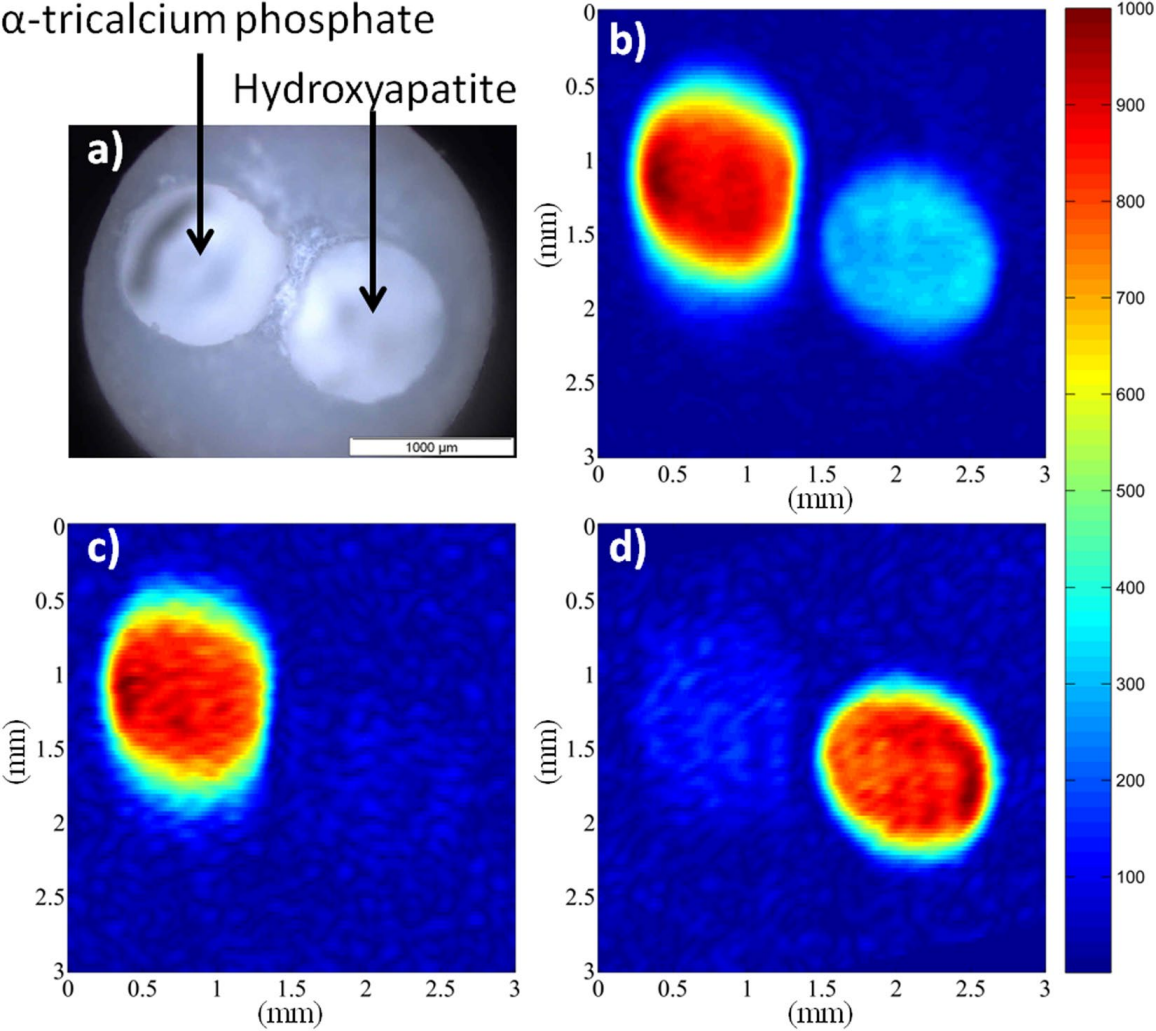
(**a**) Picture (top view) of the phantom made of a PTFE insert with two cylindrical boreholes parallel to the spinning axis (1 mm in diameter) filled with HA and α-TCP powders. (**b**) ^31^P 2D MAS MR image of the phantom (projection along the z^MAS^ axis) recorded with direct ^31^P single pulse excitation and TR = 360 s. (**c**) ^31^P 2D MAS selective image of the α-TCP phase, recorded with a ^31^P saturation comb and TR = 2 s. (**d**) ^31^P 2D CP MAS selective image of the HA phase, obtained using a CP time of 3 ms and TR = 1 s. The nominal spatial resolution for all images is 52 µm in the read dimension and 121 µm in the phase dimension (FOV = 3 × 3 mm^2^). 16 transients were co-added corresponding to an experimental time of (**b**) 51.2 hours, (**c**) 17 min and d) 8 min. The dataset was zero filled four times leading to a pixel size of 6*6 µm^2^.

It addition to the sensitivity enhancement purpose, cross-polarization also clearly provides a contrast source for imaging^27^. This is illustrated in the case of a phantom made of two cylindrical boreholes (along the rotor axis) filled respectively with hydroxyapatite (HA, Ca_10_(PO_4_)_6_(OH)_2_) and tricalcium phosphate (α-TCP, Ca_9_(PO_4_)_6_) powders (Fig. 4a), which are two biomaterials used as bone implants. As shown in Fig. 4b, the ^31^P spin density 2D image perpendicular to the z^MAS^ axis recorded with direct ^31^P excitation exhibits two spots corresponding to the HA and α-TCP cylinders. In this image recorded with a TR of 360 s, the intensity difference between the HA and α-TCP spots mainly arises from the difference between the ^31^P longitudinal relaxation times of the two phases (T_1_ ∼ 450 s for HA and ∼70 s for α-TCP). In such a case, the ^31^P signal of each phase can be obviously selected using T_1_-weighting methods. For example, a 2D ^31^P MAS image selective for α-TCP with a nominal resolution of 52 × 121 µm^2^ was obtained in 17 min using a saturation comb with a short TR of 2 s (Fig. 4c). Alternatively, ^1^H- ^31^P CPMAS can be used to selectively observe ^31^P nuclei in the vicinity of hydrogen atoms. This allows obtaining the ^31^P image selective of the HA protonated phase in a short experimental time of 8 min (Fig. 4d). This opens the way to utilize the wealth of information in NMR to generate contrast; while X-ray based imaging techniques would hardly distinguish these two biomaterials which have very similar electronic densities. Since the CP-based magnetization transfer is driven by dipolar interactions, variation of the transfer efficiency also gives information about the ^31^P local environment and mobility, which can be used for further contrast imaging. For example in the case of CDA, a significant polarization transfer between protons of water molecules and HPO_4_ group to their neighboring ^31^P atoms occurs at short CP times, while the ^1^H-^31^P transfer between hydroxyl and phosphate groups is dominant for long CP times^60^. Other solid-state MAS NMR methods based on heteronuclear dipolar interactions could also be employed for contrast purposes. For example, ^1^H-^31^P REDOR dephasing^68^ and its variants might allow attenuating the ^31^P signal of protonated phosphorus environments in the ^31^P MAS images.

**Figure 5:**
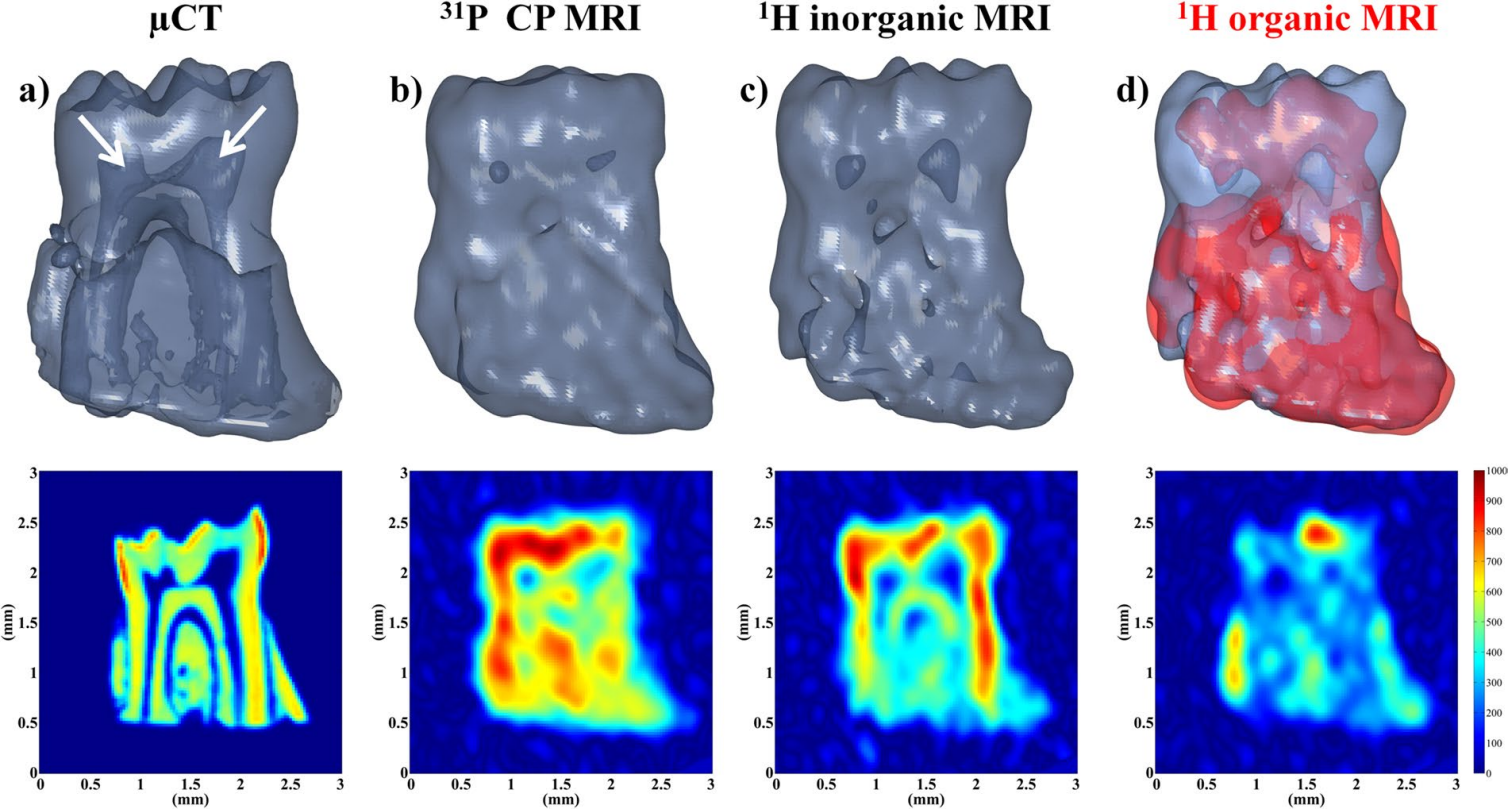
(**a**) 3D high-resolution µCT image of the mouse tooth sample (isotropic spatial resolution reduced to 27 µm). (**b**) 3D ^31^P CPMAS MR image recorded at a spinning frequency of 10 kHz using a CP contact time of 3 ms. The data were recorded with a nominal spatial resolution of 250 µm in the read dimension and 110 µm in the phase dimension which decreased to 220 µm after Hann apodization. 240 transients were co-added with a TR of 1 s corresponding to an experimental duration of 69 h. (**c**,**d**) 3D ^1^H MAS MR chemically selective images of the (**c**) OH^−^ hydroxyl groups and (**d**) aliphatic species in the mouse tooth sample. The nominal spatial resolution was 144 × 110 × 110 µm^3^. 64 transients were co-added with a TR of 1 s corresponding to an experimental duration of 18.2 h. The dataset was zero-filled 2 times leading to an isotropic voxel size of 27 µm. The 3D images are shown as isosurface representation and 2D cross-sections taken along the pulp channels of the tooth are shown below the 3D images. The white arrows indicate the location of the pulp channels.

As a proof of concept of this approach for *ex-vivo* imaging of rigid tissues, the 3D ^31^P CPMAS image of a mouse tooth (molar) attached to a piece of jaw bone was recorded at 17.6 T using a spinning frequency of 10 kHz. At this spinning rate, residual sidebands have very weak intensities and a significant increase of the ^31^P coherence lifetime is achieved (from ∼0.5 ms without spinning up to 2.4 ms) allowing the use of spin-echo sequences with acceptable signal losses. The ^31^P MAS spectrum of the mouse tooth shows a single resonance at ~3.2 ppm, characteristic of carbonated hydroxyapatite, with a linewidth of about 1.1 kHz (Figure S4 in ESI). Increasing the spinning frequency or applying ^1^H decoupling did not further reduce the ^31^P linewidth which is due to a distribution of the ^31^P isotropic chemical shift associated to local chemical disorder (inhomogeneous broadening). The 3D ^31^P image of the sample, recorded using a CPMAS spin echo sequence with a nominal spatial resolution of 250 × 220 × 220 µm^3^ and SNR of 56 for an experimental duration of 69 hours, is shown in Fig. 5b. To our knowledge, this is the best spatial resolution reported for ^31^P solid-state MRI of rigid tissues^47^. The ^31^P image nicely reflects the shape of the sample and reveals anatomic details such as the pulp channels. In the upper part of the tooth (h = 2 mm), the pulp channels are clearly observed through total absence of ^31^P intensity, and the sizes of the cavities deduced from the image (*i.e*. cross-sections of about 350 µm) correspond roughly to that measured with µCT. In the tooth roots (h = 1 mm), the channels are much thinner (section of ∼100 µm), but they remain observable through signal intensity variations. Since the image was recorded with CPMAS, only ^31^P nuclei in the vicinity of hydrogen atoms are observed. This provides contrast through the CP efficiency as a function of the phosphorus local environments and, for a contact time of 3 ms, protons of the hydroxyl groups and of adsorbed water molecules both contribute to the ^1^H-^31^P magnetization transfer. For comparison, a reference high-resolution 3D image of the sample revealing the true mineral density was recorded with micro-computed tomography (µCT, Fig. 5a). Differences in signal intensity between the cross sections of the ^31^P MRI and µCT images taken along the pulp channels suggest that the ^31^P signal of the bone component is further enhanced when compared to the tooth component. This larger signal enhancement for the bone region could be likely related to a larger content of adsorbed water molecules by contrast with the dentine part of the tooth.

The spatial resolution reached in the ^31^P CPMAS spin echo image (using a read-gradient strength of 24.86 G.cm^−1^ and rotating phase-gradient strength of 33.15 G.cm^−1^ with an equivalent rectangle duration of 0.8 ms) did not give clear evidence of the thin enamel layer observed in the µCT image because its thickness (∼60 µm) is below the spatial resolution. As already pointed out, the ^1^H-^31^P polarization transfer between hydroxyl and phosphate groups becomes dominant for long CP times, in relation with the fast dephasing of the ^1^H signal of adsorbed water molecule. Since dentine and bone are known to exhibit significant hydroxyl deficiency while the enamel composition is much closer to stoichiometric hydroxyapatite^69–71^, the use of longer contact times could allow enhancing the signal of enamel relative to that of dentine and bone. One should also mention that a ^31^P MAS image directly reflecting the quantitative phosphate distribution in the different tissues can be obtained through ^31^P direct excitation, but at the cost of a longer experimental time due to the long ^31^P longitudinal relaxation rates in the sample.

**Figure 6:**
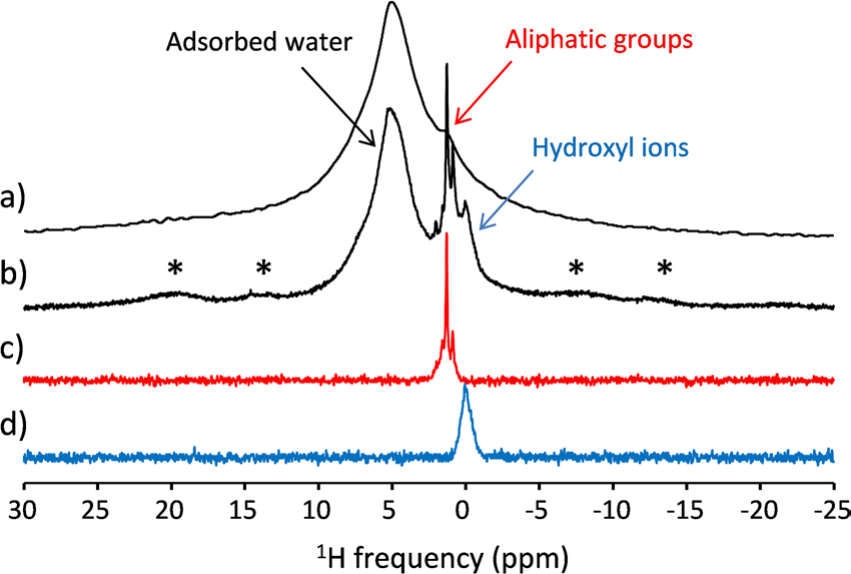
^1^H solid-state NMR spectra of the mouse tooth sample recorded at a magnetic field of 17.6 T (**a**) without sample spinning and (**b**) with MAS at a spinning frequency of 10 kHz. The asterisks indicate spinning sidebands. (**c**,**d**) ^1^H MAS selective spin echo MAS spectra of the (**c**) aliphatic protons and (**d**) hydroxyl groups, recorded with an echo time of 5.4 ms and using a refocusing R-SNOB pulse of 3 ms duration (10 kHz spinning rate).

MAS at moderate spinning frequency and high magnetic field is also well-known to significantly improve the resolution in ^1^H NMR spectra of mineralized tissues and related calcium phosphates which exhibit moderate ^1^H homonuclear dipolar interactions^56^. Indeed, the resonances of hydroxyl groups (at 0 ppm) and aliphatic protons of fats (intense peaks at 0.9, 1.3 and weaker ones at 1.6, 2.0 ppm) are clearly observed in the ^1^H MAS spectra of the mouse tooth sample, in addition to a broader line (centered at ~5.2 ppm) associated to adsorbed water molecules with a distribution of hydrogen bonding and possibly hydrogen phosphate groups. By contrast, the ^1^H spectra acquired without MAS exhibits only one broad resonance (fwhm = 3 kHz) with a shoulder at about 1 ppm and suffers from a lack of spectral resolution (see Fig. 6). As in the case of ^31^P, MAS results in a large increase of the transverse dephasing times of the ^1^H resonances of the weakly-coupled hydroxyl and aliphatic groups which rise up to about 3.6 ms and 2.2 ms respectively at spinning frequency of 10 kHz. A significant T_2_’ increase is also observed for the less strongly-coupled part of the bound-water resonance. This increase of both ^1^H spectral resolution and coherence lifetimes allows using spin echo sequences with frequency-selective soft pulse of long duration and opens the way for ^1^H chemically selective MR imaging in rigid tissues. Moreover, the sensitivity gain resulting from the use of MAS and very high magnetic field enables to record chemically-selective 3D ^1^H images reflecting the distribution of hydroxyl and aliphatic protons (corresponding to less than 8 and 7 at.% of the total hydrogen content of the sample) with a nominal spatial resolution of 144 × 110 × 110 µm^3^. As shown in Fig. 5c, the 3D map of the apatitic-OH content reveals details of the sample microstructure and evidences the pulp channels and periodontal ligament through the absence of signal intensity. The ^1^H MAS MR image exhibits higher signal intensity for the enamel part of the sample reflecting the large hydroxyl amount in the apatite crystals forming enamel, in agreement with what was reported for apatite crystals in rigid tissues^70, 71^. Further comparison with the µCT image also suggests that the apatitic-OH content is larger in dentine than in the bone part of the tooth sample. By contrast, the ^1^H image of aliphatic protons clearly reveals a much larger content of fat in bone than in dentine and enamel. It also shows an intense spot of fat in the upper part of a pulp channel which could be understood as the result of the effect of centrifugal forces induced by the sample spinning since the channels were oriented perpendicular to the spinning axis. These ^1^H chemically-selective images, recorded in about 18 hours, clearly reveal the potentialities of high field MAS MRI to provide *ex vivo* detailed 3D chemical and structural mapping in hard tissues that could be hardly obtained using other imaging methods.

## Conclusion

In this work, we have demonstrated that the combination of MAS with rotating pulsed field gradients allows overcoming difficulties associated with large line broadenings and short dephasing times in rigid solids. This approach enabled to record *ex vivo* ^31^P 3D and 2D slice-selected images of rigid tissues and biomaterials using robust spin echo sequences at very high magnetic field, with greatly improved SNR and spatial resolution when compared to static conditions. Additional selection and contrast schemes, such as dipolar dephasing or polarization transfer NMR methods, or high-resolution chemical shift imaging approaches can be tailored for additional depiction of chemical variations with spatial localization. In these materials, MAS at moderate spinning rate and very high magnetic field also provides high resolution ^1^H solid-state spectra enabling chemically selective imaging with frequency selective excitation schemes. This allowed probing selectively the 3D distribution of the apatitic hydroxyl protons or of the aliphatic ones inside a dried mouse tooth attached to the jaw bone, with an isotropic spatial resolution nearing 100 µm. The high field MAS MRI approach thus offers great potential for *ex vivo* studies in small animal models (rodents) such as hard tissues modification due to specific diseases or osteointegration of calcium phosphate implants.

## Electronic supplementary material

Supplementary Information
