# Supplementary material for: Solid-state 31P and 1H chemical MR micro-imaging of hard tissues and biomaterials with magic angle spinning at very high magnetic field
Source: Sci Rep. 2017 Aug 15;7:8224. doi: 10.1038/s41598-017-08458-0 (PMC5557955; doi:10.1038/s41598-017-08458-0)
Supplement: Supplementary file 1 — Supplementary Information [file 41598_2017_8458_MOESM1_ESM.pdf]

Solid-state ^{31}P and ^{1}H chemical MR micro-imaging of hard tissues and biomaterials with magic angle spinning at very high magnetic field

Maxime Yon[1*], Vincent Sarou-Kanian[1], Ulrich Scheler[2], Jean-Michel Bouler[3], Bruno Bujoli[3], Dominique Massiot[1], Franck Fayon[1*]

[1]CNRS, CEMHTI UPR3079, Université d'Orléans, F-45071 Orléans, France
[2]Leibniz-Institut für Polymerforschung Dresden e.V., Hohe Str. 6, Dresden, Germany
[3]CEISAM, Université de Nantes, CNRS, 2 rue de la Houssinière, BP 92208, 44322 Nantes Cedex 3, France

* Corresponding authors: maxime.yon@cnrs-orleans.fr; franck.fayon@cnrs-orleans.fr

Supplementary information

Content:

Figure S1: Scheme showing the orientation of the mouse tooth centered and held in the rotor by compacted alumina powder.

Figure S2: ^{31}P longitudinal relaxation curves of the CDA sample recorded at magnetic fields of 9.4 (black triangles) and 17.6 T (red circles) with a MAS spinning frequency of 10 kHz, using a saturation recovery experiment. The lines correspond to best fits of the experimental data to a bi-exponential recovery function, with slow- and fast-relaxing components. This behavior could be related to the presence of paramagnetic ions as impurities in the synthetic CDA sample. The ^{31}P longitudinal relaxation constants are T_1^{slow}= 15 s, T_1^{fast}= 1 s at 9.4 T, and T_1^{slow}= 22 s, T_1^{fast}= 2.5 s at 17.6 T, the slow- and fast-relaxing component having relative weights of 80 and 20 %, respectively.

Figure S3: (a) ^{31}P longitudinal relaxation curves of the mouse tooth sample recorded at magnetic fields of 9.4 (black triangles) and 17.6 T (red circles) with a MAS spinning frequency of 10 kHz, using a saturation recovery experiment. The lines correspond to best fits of the experimental data to a single-exponential recovery function. The corresponding ^{31}P longitudinal relaxation constants are T_1= 120 s at 9.4 T and T_1= 140 s at 17.6 T.

Figure S4: ^{31}P MAS NMR spectra the mouse tooth sample and the synthetic calcium-deficient hydroxyapatite (CDA) powder.

Table S1: Table of assignment, full width at half maximum (fwhm), transversal relaxation time refocused by a spin echo sequence (T_2') in static and MAS at 10 kHz and longitudinal relaxation time (T_1) in MAS at 10 kHz of the ^{1}H and ^{31}P resonances of the mouse tooth.

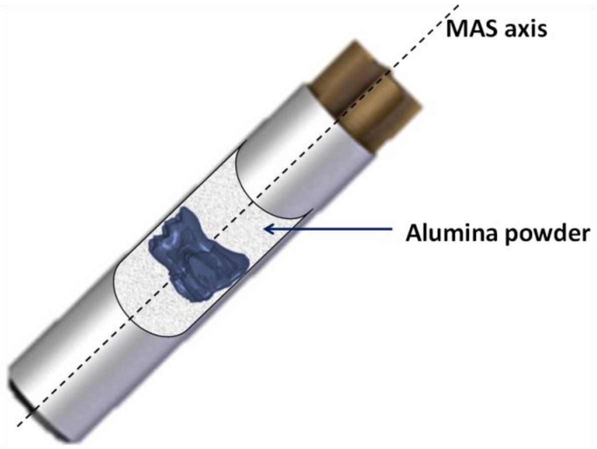

Figure S1: Scheme showing the orientation of the mouse tooth centered and held in the rotor by compacted alumina powder.

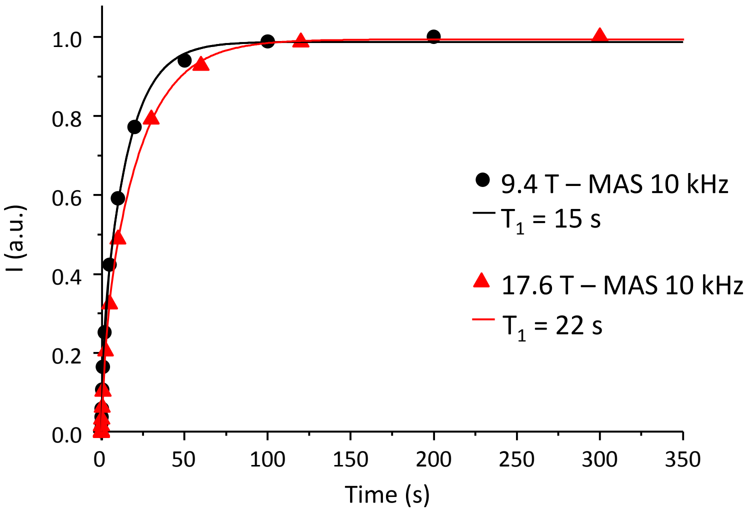

Figure S2: ^{31}P longitudinal relaxation curves of the CDA sample recorded at magnetic fields of 9.4 (black circles) and 17.6 T (red triangles) with a MAS spinning frequency of 10 kHz, using a saturation recovery experiment. The lines correspond to best fits of the experimental data to a bi-exponential recovery function, with slow- and fast-relaxing components. This behavior could be related to the presence of paramagnetic ions as impurities in the synthetic CDA sample. The ^{31}P longitudinal relaxation constants are T_1^{slow}= 15 s, T_1^{fast}= 1 s at 9.4 T, and T_1^{slow}= 22 s, T_1^{fast}= 2.5 s at 17.6 T, the slow- and fast-relaxing component at both field strengths having relative weights of 80 and 20 %, respectively.

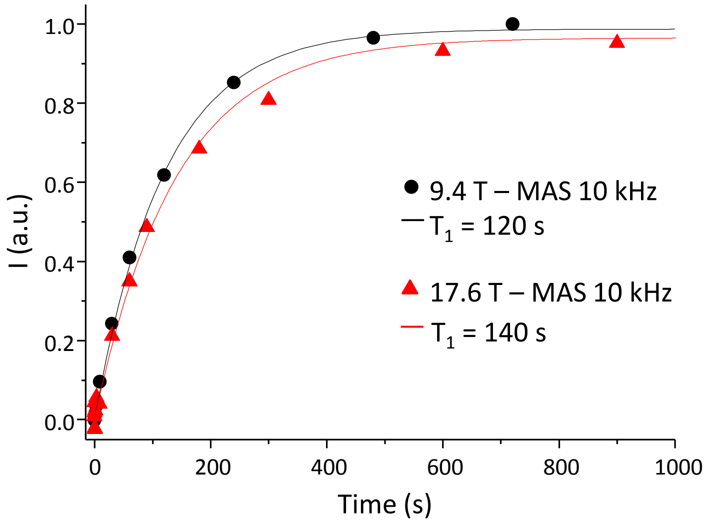

Figure S3: (a) ^{31}P longitudinal relaxation curves of the mouse tooth sample recorded at magnetic fields of 9.4 (black triangles) and 17.6 T (red circles) with a MAS spinning frequency of 10 kHz, using a saturation recovery experiment. The lines correspond to best fits of the experimental data to a single-exponential recovery function. The corresponding ^{31}P longitudinal relaxation constants are T_1= 120 s at 9.4 T and T_1= 140 s at 17.6 T.

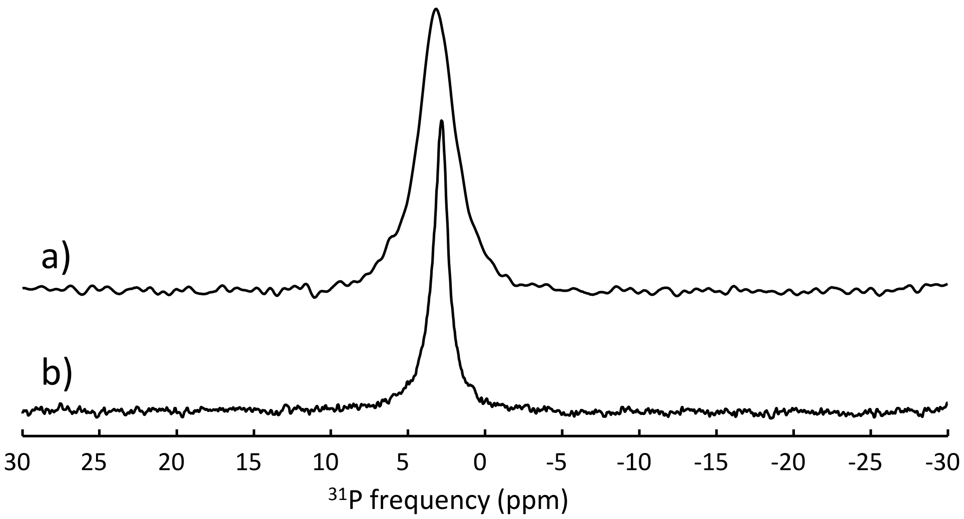

Figure S4: ^{31}P MAS NMR spectra of a) the mouse tooth sample and b) the synthetic calcium-deficient hydroxyapatite (CDA) powder, recorded at 17.6 T using a spinning frequency of 10 kHz. Both spectra show a single resonance with a ^{31}P isotopic chemical shift of a) 2.8 and b) 3.2 ppm and full width at half maximum (fwhm) of 1100 and 340 Hz.

Assignments	δ_{ISO} (ppm)	Fwhm (Hz)	T_2' static (ms)	T_2' MAS 10 kHz (ms)	T_1 MAS 10 kHz (s)
OH^- hydroxyl	0.0	800	-	3.65	0.75
CH_2 aliphatic	1.3	170	-	2.37	0.68
CH_3 aliphatic	0.9	140	-	2.16	0.68
Adsorbed H_2O	5.1	2100	0.16	0.22	0.92
^{31}P hydroxyapatite	3.2	1100	0.55	2.43	140

Table S1: Table of assignment, isotropic chemical shift (δ_{ISO}), full width at half maximum (fwhm), transversal dephasing time (T_2') under static and MAS (spinning frequency of 10 kHz) conditions and longitudinal relaxation time (T_1) at 10 kHz MAS frequency of the ^1H and ^{31}P resonances of the mouse tooth sample. T2' values were determined from fits to single exponential function of the intensity decay as function of the echo time in a Hahn echo experiment. Longitudinal relaxation rates were measured using a saturation-recovery sequence.
